# Silver-Based Plasmonic Nanoparticles for and Their Use in Biosensing

**DOI:** 10.3390/bios9020078

**Published:** 2019-06-10

**Authors:** Alexis Loiseau, Victoire Asila, Gabriel Boitel-Aullen, Mylan Lam, Michèle Salmain, Souhir Boujday

**Affiliations:** 1 Laboratoire de Réactivité de Surface (LRS), Sorbonne Université, CNRS, UMR 7197, 4 Place Jussieu, F-75005 Paris, France; alexis.loiseau@sorbonne-universite.fr; 2 Sorbonne Université, Faculté des Sciences et Ingénierie, Master de Chimie, Profil MatNanoBio, 4 Place Jussieu, F-75005 Paris, France; victoire.asila@upmc.fr (V.A.); g.boitelaullen@gmail.com (G.B.-A.); mylan951@hotmail.fr (M.L.); 3 Institut Parisien de Chimie Moléculaire (IPCM), Sorbonne Université, CNRS, 4 Place Jussieu, F-75005 Paris, France; michele.salmain@sorbonne-universite.fr

**Keywords:** silver nanoparticles, synthesis, coating, alloy, core@shell, LSPR, biosensors

## Abstract

The localized surface plasmon resonance (LSPR) property of metallic nanoparticles is widely exploited for chemical and biological sensing. Selective biosensing of molecules using functionalized nanoparticles has become a major research interdisciplinary area between chemistry, biology and material science. Noble metals, especially gold (Au) and silver (Ag) nanoparticles, exhibit unique and tunable plasmonic properties; the control over these metal nanostructures size and shape allows manipulating their LSPR and their response to the local environment. In this review, we will focus on Ag-based nanoparticles, a metal that has probably played the most important role in the development of the latest plasmonic applications, owing to its unique properties. We will first browse the methods for AgNPs synthesis allowing for controlled size, uniformity and shape. Ag-based biosensing is often performed with coated particles; therefore, in a second part, we will explore various coating strategies (organics, polymers, and inorganics) and their influence on coated-AgNPs properties. The third part will be devoted to the combination of gold and silver for plasmonic biosensing, in particular the use of mixed Ag and AuNPs, i.e., AgAu alloys or Ag-Au core@shell nanoparticles will be outlined. In the last part, selected examples of Ag and AgAu-based plasmonic biosensors will be presented.

## 1. Introduction

The first use of silver (Ag) as an antimicrobial and antibacterial agent goes back to the ancient Greek and Roman Empire [1,2]. At that time, the medicinal and preservative properties of silver were mainly used to protect vessels from bacterial attacks and to make water and other liquids potable [1,3,4,5]. Globally, it was already known to be an efficient weapon against the growth of pathogen factors [6]. The antimicrobial effect of silver arises from the interaction of silver ions with thiol groups of vital bacterial enzymes and proteins that lead to cell death [4,5,7]. Over the past decades, silver has been engineered into nanoparticles (NPs) (at least one dimension is smaller than 100 nm) [8]. Although metallic NPs have been present in artefacts for a very long time [9,10], as attested by medieval stained glasses, and even earlier as for the Lycurgus Cup of the British Museum in London, dated from the 5th century, scientific knowledge about NPs is quite recent [11]. Synthesis of metallic NPs can be achieved according to two distinct nanofabrication methods. On the one hand, top-down approaches involve physical processes such as lithography or chemical processes controlled by external experimental parameters to create nanoscale structures starting from larger dimensions to the nanometer range [12]. This can be achieved by milling or high pressure homogenization [13]. On the other hand, the bottom-up approaches use atoms or small molecules as the building blocks of multi-level structures to build up more complex nanoscale assemblies or directed self-assemblies that perform various operations [14]. This method is extremely valuable since it is free of waste or unused materials [15]. This can be achieved by controlled precipitation (or crystallization) and evaporation from a precursor [13,16]. Generally, top-down techniques produce NPs that are mostly crystalline but high energy or pressure is required to achieve nanometer range comminution, which may also lead to contamination if a milling medium is used. In contrast, bottom-up processes involve dissolution, followed by precipitation or drying. The mechanical energy input is thus minimal, and the resulting NPs can be crystalline or amorphous, depending on the synthesis conditions. Metallic NPs feature unique physical properties such as high ratio-to-surface area and volume. Moreover, the confinement effect confers reactivity as well as mechanical, electromagnetic, chemical and optical properties that differ from those of the bulk metals [17,18,19]. Indeed, particles properties change drastically at the nanometer scale [11]. Metallic NPs find applications in various fields, from catalysis to the detection of biological molecules in solution [8,20]. In the biomedical field, they can be used in drug delivery, photothermal therapy, or imaging [21,22,23,24,25]. In what follows, we will focus the use of NPs for biosensing applications.

Among all metallic NPs, gold (Au) and silver (Ag) nanoparticles exhibit the most interesting physical properties for biosensing [26,27]. Even if gold nanoparticles (AuNPs) remain the most studied for this application area because of their good chemical stability and biocompatibility [20,28,29], silver nanoparticles (AgNPs) offer better results in terms of sensitivity [30]. One of the most characteristic physical properties of metallic NPs is the localized surface plasmon resonance (LSPR), which is responsible for the bright color of the nanoparticle colloidal suspensions [26,27,31,32,33,34]. Indeed, AuNPs and AgNPs are specifically investigated for their optical properties thanks to their strong interactions with light [35,36,37,38,39,40]. The electrons at the surface of the metallic NPs undergo a collective oscillation when irradiated at a specific wavelength, called surface plasmon resonance (SPR), resulting in the appearance of the electromagnetic field localized on the NPs [30,31,41]. When the oscillations of the electromagnetic field of an incident electromagnetic wave are in resonance with those of the local electromagnetic field of the NPs, the LSPR phenomenon occurs, which is characterized by the resonance oscillation frequencies. Thus, LSPR is the consequence of the confinement of the electric field within a small metallic sphere whose radius is much smaller than the wavelength [10]. This property can be tuned by controlling parameters such as shape, size, uniformity and surface coating [27,31] and is often used for biosensing applications in the field of biology, biomedicine and biochemistry [42]. For this purpose, AgNPs of different shapes and sizes, from the simplest to the most sophisticated, can be readily obtained thanks to the large range of techniques now available that will be presented later in this review for a conceptual opportunity of biosensing. Owing to their plasmonic properties, metallic nanoparticles are also responsible for enhancing Raman scattering of molecules adsorbed at their surface, giving rise to the so-called surface enhanced Raman spectroscopy (SERS) [32,43], a powerful vibrational spectroscopy with impressive enhancement factors of up to 14–15 orders of magnitude [44]. It is worthy to note that this phenomenon is very different from propagative SPR or surface plasmon polariton (SPP) that occurs at the plane surface of large metallic structures, or on metallic nanowires, on which one direction is regarded as infinite. Colloidal suspensions of small spherical AuNPs (10 nm diameter) are red-colored and display an absorption band at 520 nm, while similar AgNPs are yellow and absorb around 380 nm [45]. As these two absorption bands are in the visible part of the electromagnetic spectrum, they allow a colorimetric detection of biomolecules by inducing changes in the position (and possibly in the intensity) of the LSPR band.

As stated above, the position of the LSPR band of AuNPs and AgNPs depends on their size, uniformity, shape, dispersion, composition (ratio Au:Ag), and also on the dielectric constant of the surrounding medium [9,26,30,32,43,46,47]. Therefore, modifying any of these parameters induces wavelength shifts. For example, size increase shifts the LSPR band to higher wavelength, i.e., red-shifted [47,48]; NPs aggregation also induces a red-shift. While isotropic (spherical) NPs have a unique absorption band, anisotropic ones can display several absorption bands [47,48]. Two formats are typically encountered for colorimetric and plasmonic biosensors, i.e., aggregation-based assays [49,50,51] and LSPR-based ones [38]. The refractive index sensitivity (RIS), expressed in nm/refractive index unit (RIU) (nanometer per refractive index unit) is a measure of the shift in wavelength of the LSPR peak: the more the peak is shifted for small variations of refractive index (RI), the more sensitive the biosensor is (i.e., the highest the sensitivity is). AgNPs are described as more sensitive than AuNPs for the second biosensing strategy [52]. Indeed, a study showed that the RIS for AgNPs and AuNPs increased from 153 to 265 nm/RIU and 128 to 233 nm/RIU, respectively, for sizes 5 to 50 nm [53]. However, combining the two metals is very attractive and offers a wide range of possibilities.

In what follows we discuss multiple strategies to produce AgNPs of various sizes and shapes that are both key factors contributing to the modulation of major optical properties. Then, we will explore different coatings (organic, polymer, inorganic) on silver nanoparticles and highlight the influence of AgNPs coating process on their fate, stability, and toxicity in a given environment as well as their properties regarding plasmonic biosensing applications. We will also cover the use of mixed Ag and Au NPs (i.e., AgAu alloy and Ag-Au core@shell structures) for optical biosensing. Finally, we will present selected examples of Ag and AgAu-based plasmonic biosensors and highlight the merit of silver-containing nanoparticles in this area.

## 2. Engineering Silver Nanoparticles for Biosensing: Shape-Properties Correlation

Much less effort has been put on the use of AgNPs for biosensors compared to AuNPs. Indeed, 85,570 papers are referenced in the SciFinder™ database with the keyword “gold nanoparticles” compared to 63,770 for “silver nanoparticles”. Combined with the keyword “plasmonic biosensors”, these numbers are reduced to 424 and 112 papers, for AuNPs and AgNPs, respectively.

The synthesis of AgNPs is achieved through chemical, physical, or biological strategies. The review written by B. Khodashenas and H. R. Ghorbani in 2015, summarizes the wide range of synthetic methods reported to date as a function of the desired nanoparticle’s shape [54]. We summarize in Table 1 the main strategies employed for AgNPs engineering and the related size, shape, and applications.

### 2.1. AgNPs Synthesis by Chemical Reduction Using Citrate and/or Ascorbate

**Figure 1:**
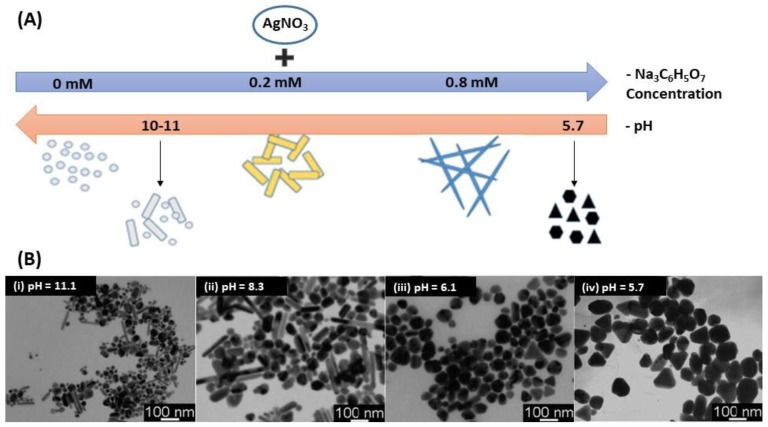
AgNPs synthesis using citrate: (**A**) Experimental conditions affecting the silver nanoparticle AgNPs shape and (**B**) TEM images of the AgNPs synthesized at different pH values: (i) 11.1, (ii) 8.3, (iii) 6.1 and (iv) 5.7. Adapted from [72]. Copyright (2009), American Chemical Society.

Nowadays, among the wide range of synthetic methods, the chemical reduction by the bottom-up approach is the most common method to prepare AgNPs. The reaction is performed in either an aqueous or an organic solvent. The commonly used method is inspired from the Turkevich synthesis for AuNPs [67], and was first introduced by Lee’s group in 1982 [68]. It involves a silver precursor, usually an inorganic compound such as silver nitrate (AgNO_3_) that liberates silver ions (Ag^+^) in solution, then, trisodium citrate (Na_3_C_6_H_5_O_7_) reduces silver and further stabilizes the resulting AgNPs [69]. However, this method often leads to polydisperse nanoparticles and several works attempted to reduce the dispersity [69,70]. Indeed, many factors have been demonstrated to play an important role on the size, shape and color of NPs such as temperature and acidity of the solution [71]. Actually, the acidity of the solution has a strong influence on the AgNPs’ shape. In 2009, Dong et al. showed that the shape of AgNPs was significantly influenced by the pH using citrate as a reducing as well as stabilizing agent (Figure 1A) [72]. They found out that the shape of AgNPs at a high pH was a mixture of spherical and rod-like particles while the predominant shapes were triangular and polygonal particles at low pH (Figure 1B) [71,72].

**Figure 2:**
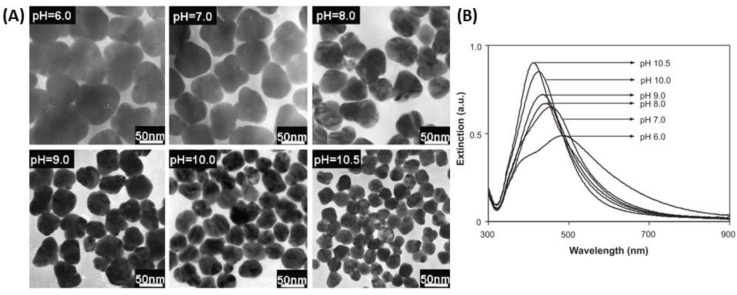
(**A**) TEM images and (**B**) UV-vis spectra of the AgNPs prepared at pH 6.0, 7.0, 8.0, 9.0, 10.0 and 10.5 by using ascorbate as reductant. Adapted from [73]. Copyright (2010), Elsevier B.V. All rights reserved.

This observation was confirmed by Qin et al. in 2010 as more spherical particles were obtained when the pH was increased by using ascorbate as a reductant and citrate as a stabilizer [73]. Furthermore, they demonstrated that NP size varied as a function of pH. Indeed, AgNPs were smaller due to the increased reducing activity of citrate or ascorbic acid (AA) when the pH was increased that in turn decreased the number of nuclei [71,73]. These studies also showed that the type of reductant affects the shape of the AgNPs due to the pH-dependent redox potential and thus the pH-dependent reduction rate of the precursor (Ag^+^). A more precise adjustment of the equilibrium between nucleation and growth allows better control over the shape of AgNPs. The use of ascorbic acid as reducing agent tends to afford spherical particles over the pH range between 6 and 11 conversely to citrate (Figure 2).

Another study showed that the position of the LSPR band shifted as a function of the acidity [71]. Indeed, the LSPR band intensity increased and the band blue-shifted as the pH increased. The bands became sharper, and the nanoparticles size decreased accordingly as demonstrated by the TEM images in Figure 2. Finally, citrate was also used as reducing agent to form Ag nanoshell (AgNS) on a silica sphere [74,75,76]. The thickness of the silver shell could be tailored by varying the number of deposition cycles. Such a weak reducing agent was a prerequisite to the growth of a silver shell, so that the Ag seeds grew only in size during the Ag reduction whereas no new nucleation centers were introduced, which ensured the minimal amount of Ag colloids in the suspension accompanying the AgNS growth.

### 2.2. AgNPs Synthesis: Anisotropic Shapes

Investigations on anisotropic shapes and morphologies of NPs have increased during the last decade, most often relying on the development of seed-mediated synthetic methods [77]. Such syntheses of anisotropic NPs have attracted interest because the structural, optical, electronic, magnetic, and catalytic properties are different from, and most often superior to, those of spherical NPs for a comparable size [78]. In particular, the most striking properties of anisotropic and hollow NPs lie in the appearance of a plasmon band at a longer wavelength (near-infrared region) than that of spherical NPs [79,80]. Different shapes and sizes of AgNPs can be synthesized thanks to the large range of techniques now available [72,77,81,82,83,84,85]. Inspired by gold nanorods synthesis, silver nanorods (AgNRs) were synthesized following a process involving the reduction of AgNO_3_ with sodium borohydride (NaBH_4_) in the presence of citrate followed by growth of seeds into NRs in the presence of AA and cetyltrimethylammonium bromide (CTAB) [86]. In 2011, Zaheer and Rafiuddin achieved the synthesis of flower-like silver NPs at room temperature by a wet chemical reduction strategy [87]. It involves the use of AA as reducing agent and CTAB. Such a shape is the result of the aggregation of small NPls and NRs. Flower-like AgNPs were used as SERS substrates and showed high sensitivity to rhodamine 6G.

**Figure 3:**
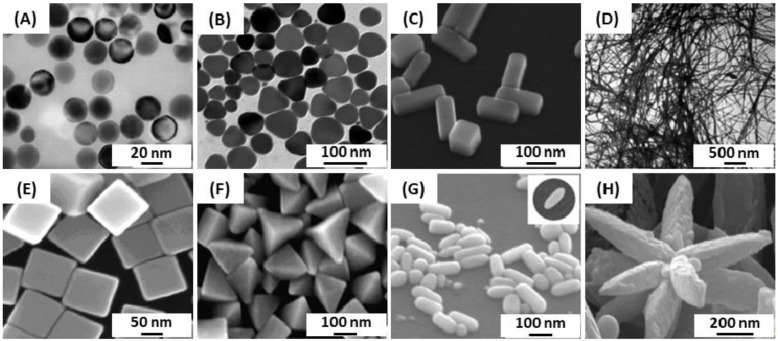
TEM images of silver nanoparticles with different shapes: (**A**) nanospheres, (**B**) nanoprisms, (**C**) nanobars and (**D**) nanowires. SEM images of (**E**) nanocubes, (**F**) pyramids, (**G**) nanorice and (**H**) nanoflowers. Adapted from [54,56]. Copyright (2009), Springer Science Business Media, LLC.

Several other studies showed that it is possible to make the transition from spherical to nanowire particles through nanorods by modulating the experimental conditions such as temperature, Ag precursor concentration, pH of the solution, reducing agent (citric acid, AA, and NaBH_4_), and the method (chemical, physical or biological) (Figure 3) [54,56,88].

**Figure 4:**
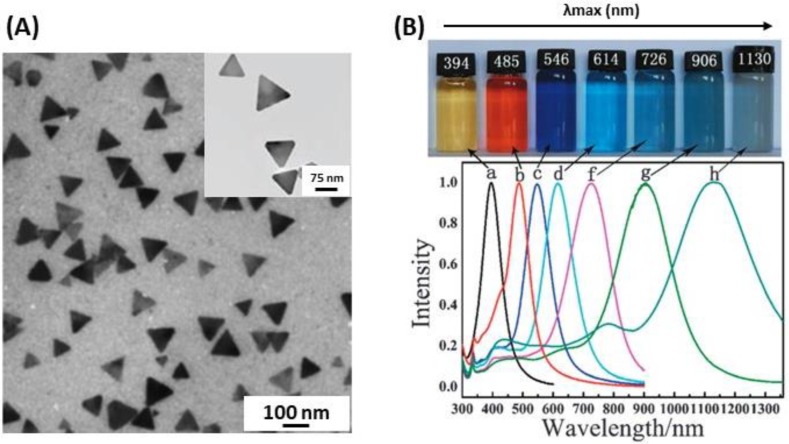
(**A**) TEM image of AgNPls. (**B**) Dispersions of Ag (a) sphere and (b–h) nanoplate colloids with different colors and corresponding UV-vis-NIR extinction spectra that reflect the ability to tune the plasmon resonance of the nanoplates across the visible near-infrared (NIR) portion of the spectrum (500–1100 nm). The nanoplate optical resonance and size are tuned according to different rounds of Ag seed growth. Adapted from [90,93]. Copyright (2008, 2013), Royal Society of Chemistry.

A particularly interesting morphology for the development of LSPR biosensors is that of silver nanoplates (AgNPls), in which the lateral dimensions are larger than their height, so that they possess an extremely high degree of anisotropy (Figure 4A). Although the systems used for LSPR biosensing have been mainly ordered arrays of triangular NPls (TNPls) prepared by nanosphere lithography (NSL), wet chemistry techniques are one of the most widely used methods with a tight control of size and shape [30,89,90]. The wet chemistry approaches to synthesize AgNPls are light-mediated methods that relate to the use of visible light as a driving force for the oriented attachment of preformed nanoparticles. Some chemical reduction methods are based on the reduction of Ag^+^ on Ag seeds using a weakly reducing agent (citrate or ascorbate), in the presence of CTAB, by analogy to the well-known growth of Au nanorods [90,91]. AgNPls optical resonance can be tuned from 500 to 1100 nm by precisely controlling the plate diameter and thickness [92]. AgNPls have extremely large absorbing and scattering cross-sections across the visible and near-infrared (NIR) regions (Figure 4B) [93]; they have applications in SERS, photovoltaics, molecular detection, and photothermal-based therapies [30,89,94].

**Figure 5:**
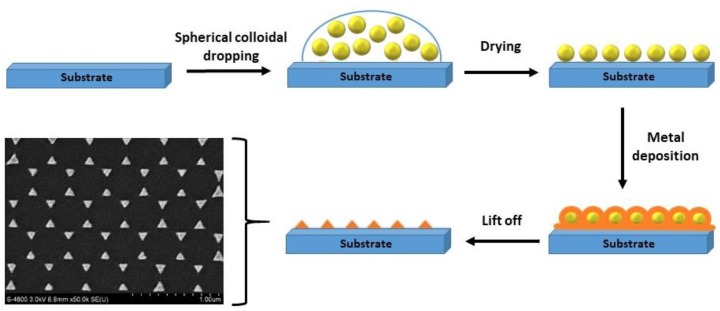
Diagram of the nanosphere lithography (NSL) mechanism. SEM image of topography of the triangular Ag nanoparticles fabricated by NSL. Adapted from [96]. Copyright (2011), publisher and licensee Dove Medical Press Ltd.

Nanoprisms (NPrs) seem to present a classic triangular shape, but a closer observation showed that the triangles apexes were flat. Compared to spherical nanoparticles, NPrs with flat apexes and (111) crystal planes, exhibited greater antibacterial property [95]. Ag triangular nanoparticles may be also fabricated by NSL. In fact, Haes and Duyne demonstrated a very good RIS by tuning the shape and the size, and a short-range, sensing length scale determined by the characteristic decay length of the local electromagnetic field [57]. NSL is widely used to get mono-disperse, surface-confined Ag triangular NPs. It is based on the creation of a single layer crystal nanosphere mask with a suspension containing monodisperse spherical colloids (polystyrene) onto a glass substrate [96,97]. Then, a drying step is required, and the mask is formed. After that, a film of silver material is deposited over the support and the mask is then removed by a step of sonication in an adequate solvent, as shown in Figure 5. The size of the nanotriangles is controlled by the diameter of the nanospheres deposited [97].

### 2.3. AgNPs Synthesis: Chemical Reduction Using Unconventional Ligands

**Figure 6:**
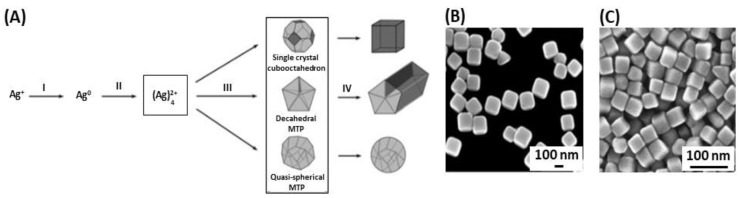
(**A**) illustration of (I) Ag^+^ reduction by polyol process; (II) formation of Ag clusters; (III) seed nucleation; and (IV) seed growth into nanocubes, nanorods or nanowires, and nanospheres. SEM images of Ag nanocubes synthesized by mixing AgNO_3_ and polyvinyl pyrrolidone (PVP) *via* polyol process: (**B**) without and (**C**) sulfide-assisted synthesis (reaction time: 45 min vs. 7 min, respectively). Adapted from [98,102]. Copyright (2004), © WILEY-VCH Verlag GmbH & Co. KGaA, Weinheim. Copyright (2006), Elsevier B.V. All rights reserved.

In 2002, it was reported that silver nanocubes were synthesized with a polyol process (Figure 6A), which involves the reduction of silver thanks to the hydroxyl groups of ethylene glycol [82]. The alcohol acts both as solvent and reducing agent [54]. A capping agent is then added; generally polyvinyl pyrrolidone (PVP) whose role is to build the cubic shape of the NPs [98,99,100]. Later, Tao et al. (2006) found that the ratio between Ag^+^ and the number of repeating units of PVP defined the geometry of the NPs [101]. The nanocube formation was favorable when the ratio was high. On the contrary, the nanowire geometry would have been favorable. In the same year, Siekkinen et al. found out that adding a small quantity of sodium sulfide (Na_2_S) or sodium hydrosulfide (NaHS) speeds up the reaction, from 16–26 min to 3–8 min (Figure 6B,C) [102].

**Figure 7:**
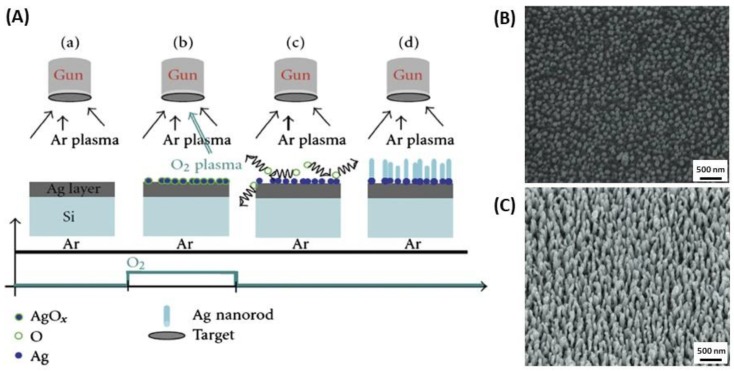
(**A**) Synthesis of silver nanorods by sputtering process: oxidation reduction growth (ORG). SEM images of (**B**) Ag nuclei and (**C**) AgNRs arrays. Adapted from [64].

AgNRs were synthesized by using a method called oxidation reduction growth (ORG) [64]. Firstly, a thin silver film is deposited on a silica surface with a relatively constant flow of argon gas. Then, silver oxide seeds are formed during sputtering. In the sputtering process, the temperature increased to reach 200 to 300 °C. Hence, silver oxide dissolved and released oxygen. It allowed silver nanorods to grow without oxygen (Figure 7).

## 3. Coating of Silver Nanoparticles

There are very few studies on bare AgNPs as plasmonic biosensors. One of the reasons concerns their toxicity, even if most biosensors operate ex vivo. AgNPs toxicity was extensively described in a book published in 2019 [103]. The second reason, and most probably the major limitation for bare AgNPs use in biosensing, is their poor stability and less straightforward surface chemistry [104,105]. To overcome these limitations, AgNPs were coated by a large variety of compounds; the coating process has a marked influence on the fate, stability, and toxicity of AgNPs in a given environment [106,107,108]. The coating of the NPs provides electrostatic, steric, or electrosteric repulsive forces between particles, which allows them to resist aggregation phenomena [105]. In the literature, various coating methods have been explored to cover AgNPs with an organic or an inorganic shell and highlighted the interest of coating AgNPs for plasmonic biosensing applications (Table 2). Hence, both the nature of the coating reactant and the thickness of the coating layer have a decisive influence on the optical properties of the NPs. In what follows, we will present examples of AgNPs coatings and discuss their influence regarding plasmonic biosensing. Interest will be first brought to organic-coated (excluding polymers) AgNPs, then to polymer-coated ones in order to improve electrostatic, steric and electrosteric stabilization of AgNPs. Finally, a brief overview of silica coating on NPs will be made.

### 3.1. Organic Coatings

**Figure 8:**
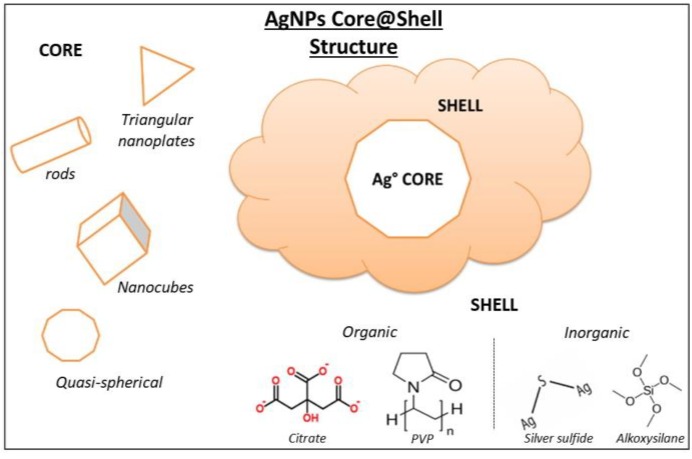
Silver nanoparticles core@shell structure. Adapted from [114]. Copyright (2012), American Chemical Society.

AgNPs synthesis typically uses organic compounds to promote stabilization and prevent aggregation of the particles by adsorption or covalent attachment to the particles surface. In the literature, they are often referred to as “capping agents” when they are applied during synthesis. It was proven that they have an effect on the size and shape control of the AgNPs [98]. Therefore, the function of organic coating in the stabilization and the growth of AgNPs is clearly essential for their further properties [107]. There are different possible shapes of AgNPs including quasi-spheres, nanotubes, rods, or triangular nanoplates (TNPls) which also means different coating methods with capping agents of various chemical nature (Figure 8) [114].

**Figure 9:**
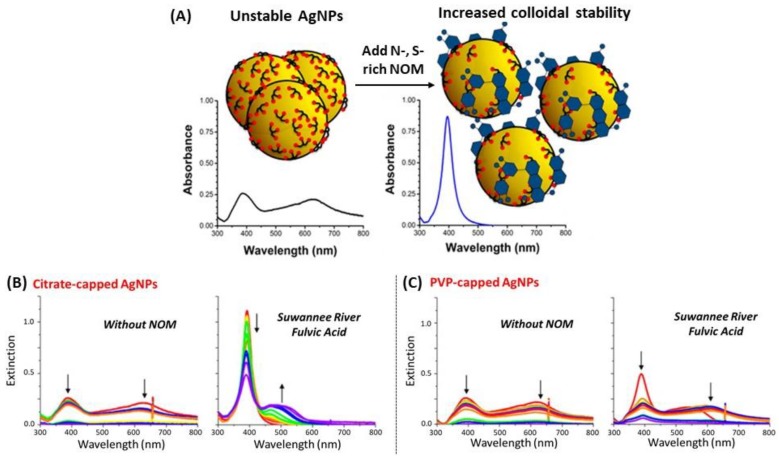
(**A**) Illustration of natural organic matter (NOM) interactions with the surface of silver nanoparticles according to the NOM’s chemical composition and the affinity of the capping agent for the AgNP surface. Colloidal stability of (**B**) citrate- and (**C**) PVP-capped AgNPs in the absence or the presence of NOM from various origins. Adapted from [117]. Copyright (2015), American Chemical Society.

Natural organic matter (NOM) is a quite interesting example of organic coating of AgNPs [115,116]; NOM significantly influenced the stability and the surface properties of NPs, and had in turn a direct effect on the transport and the AgNPs toxicity in aqueous systems. In 2015, Gunsolus and his co-workers used NOM to stabilize citrate- and PVP-capped AgNPs against aggregation [117]. AgNPs incubated with NOM showed higher primary extinction peak intensity, which means a larger population of monodisperse particles, and slower aggregate formation by observing the secondary extinction peak (Figure 9). However, we could find no example of use of NOM-coated AgNPs as LSPR biosensors, possibly because the NOM shell is too large (up to 150 nm in Reference [117]) and, as LSPR is a short distance effect, the molecular phenomena occurring at the NOM shell no longer affect the LSPR signal. Besides, another green method was used to synthesize organic-coated AgNPs from extracts of soap-root plant as stabilizers and manna of hedysarum plant as reducer [118].

**Figure 10:**
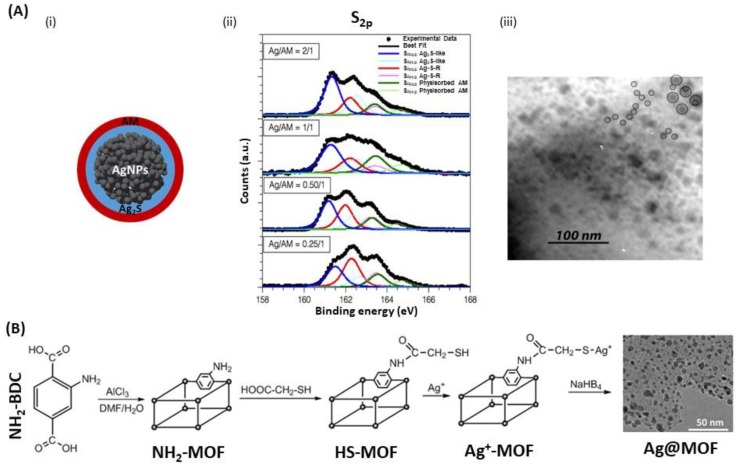
(**A**) Allylmercaptane-stabilized AgNPs: (i) core@shell morphology for allylmercaptane- (AM)-functionalized AgNPs through Ag-S chemical bonds to form the external layer, (ii) XPS spectra of Ag@AM with four different Ag/thiol ratios, and (iii) TEM images of AgNPs with Ag/AM molar ratio equal to 2/1 (AgNPs dimensions are 9 ± 3 nm and a population of NPs aggregated of 18 ± 6 nm. (**B**) Illustration of the encapsulation of AgNPs in thiol-modified metal-organic framework (MOF) as a host matrix. Adapted from [119,120]. Copyright (2012), American Chemical Society. Copyright (2015), Royal Society of Chemistry.

Many studies investigated the use of thiol-capping agents as anchoring groups for stabilizing and protecting AgNPs [108,119,120]. The thiol-capping agents are grafted to the AgNPs surface through Ag-S chemical bonds to form the external layer suggesting a core@shell morphology with an Ag core surrounded by Ag_2_S-like shell. A study showed that stabilized AgNPs, by the organic thiol, allylmercaptane (AM), were synthesized with different Ag/S molar ratios in the presence of tetraoctylammonium bromide (TOAB) and NaBH_4_ [119] *via* modified Brust−Schriffin method [121]. It has been shown that the increase in Ag/AM ratio led to an increase of the Ag_2_S layer thickness, and thus larger AgNPs were obtained, while the external AM layer remained unchanged (Figure 10A) [119]. Desireddy et al. prepared ultrastable AgNPs with a uniform size from the reduction of soluble precursor, which uses a protecting shell of *p*-mercaptobenzoic acid in semi-aqueous solution in the presence of NaBH_4_ and a coordinating solvent. This approach showed better results regarding the stability, purity and yield in very large quantities compared to those for AuNPs, due to an efficient stabilization mechanism [108]. Another approach was used by Cheng et al. using thiol-modified metal-organic framework (MOF) [120]. Herein, MOF was used as a host matrix to obtain AgNPs by using the stabilization ability of the thiol group to prevent further aggregation (Figure 10B). By controlling the initial loading amount of silver ions in the cages of thiol-MOF, monodispersed AgNPs were encapsulated in frameworks by reducing Ag^+^ with NaBH_4_, while adjusting sizes of particles from 5.3 nm to 3.9 nm, which is difficult to achieve for AgNPs because of their strong tendency to aggregate.

### 3.2. Polymer Coatings

Polymers are molecules that can adopt various conformations in solution. The chain swelling can be modulated by the temperature [122]. This aspect of polymers properties has been investigated because the main interest of polymer coating comes from steric interactions. Indeed, polymers, either grafted or adsorbed on NPs, promote dispersity in the NPs solution [123]. It has long been established that polymers with a large molecular weight and a high grafting density tend to increase the colloidal stability [124,125].

**Figure 11:**
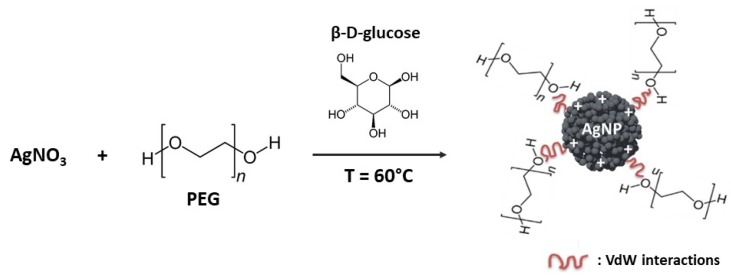
Poly(ethylene) glycol (PEG) coated method of silver nanoparticles. Inspired from [130].

Poly(ethylene) glycol (PEG) is one of the most studied polymers as stabilizing or coating agent for NPs [123,126,127]. This neutral, hydrophilic and biocompatible polymer has been approved by the Food and Drug Administration (FDA) for biomedical and pharmaceutical applications [128]. PEG improves the AgNPs dispersity in physiological conditions by steric hindrance and prevents nanoparticles aggregation [107,115,129]. Figure 11 represents only one of many ways of PEG coating by a green method [130]. Colloidal stabilization for PEG-coated AgNPs probably occurs thanks to the presence of VdW interactions:

Ag^+^_(aq)_ + PEG_(aq)_ → [Ag(PEG)]^+^_(aq)_

2 [Ag(PEG)]^+^_(aq)_ + CH_2_OH(CHOH)_4_CHO → 2 [Ag(PEG)]_(s)_ + CH_2_OH(CHOH)_4_COOH

Another polymer, called chitosan, is widely used to coat NPs because of its good biocompatibility [131,132]. It shows a good affinity for the Ag surface and confers a high stability and dispersibility to the AgNPs [91,133]. It also shows shape-directing properties by influencing the shape of the particles from spherical to triangular.

### 3.3. Silica Coating

**Figure 12:**
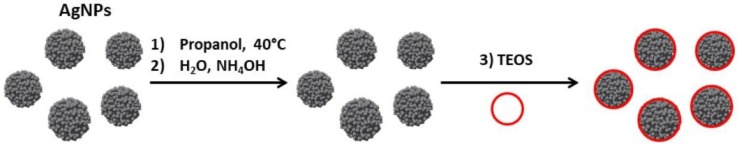
Modified Stöber method for coating of AgNPs with silica.

Among all coating materials used for plasmonic nanoparticles capping, silica occupies a pro-eminent position for multiple reasons [134,135,136]; first, silica provides a biocompatible protective shell, tunable in thickness, preventing aggregation due to electrostatic repulsion and stable in numerous solvents; second, silica synthesis is largely mastered especially through sol-gel and/or Stöber process to achieve a nanometric control of the thickness, the porosity, and the homogeneity; lastly, the presence of silanol groups on silica surface simplify the further chemical modification to introduce various surface functionalities (e.g., COOH, CHO, NH_2_, or NCO) with readily available coupling agents [137,138]. The Mie theory already predicts effects of silica shell thickness on NPs optical properties in various solvents [139]. There are several ways to coat NPs with silica, among which the modified Stöber process that enables to control the shell growth over a short time period (Figure 12) [135,140,141].

**Figure 13:**
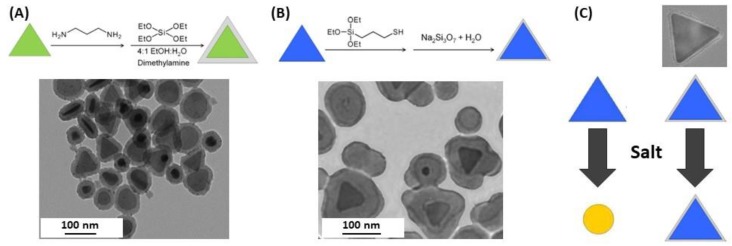
Silica coating of Ag triangular nanoplates by (**A**) diaminopropane priming and (**B**) (3-Mercaptopropyl)triethoxysilane (MPTES) priming followed by deposition from Na_2_Si_3_O_7_ solution. (**C**) The silica shell using MPTES on triangular nanoplates (AgTNPls) allows withstanding salts without adversely affecting refractive index (RI) sensitivity in relation to original uncoated particles. Adapted from [113]. Copyright (2013), Elsevier Inc. All rights reserved.

Kobayashi, Liz-Marzán and their co-workers synthesized SiO_2_-coated AgNPs by sol-gel reaction of tetraethyl orthosilicate (TEOS) [140]. They observed that the shell thickness was controlled through TEOS concentration and observed an increasing red-shift of the LSPR band for thicknesses in the range 28 to 48 nm. Larger silica shell thicknesses, 57–76 nm, induced a blue-shift of the plasmon band as well as a decrease of its intensity, which means that larger silica shells promote significant scattering at shorter wavelengths. Their findings were consistent with the theoretical spectra calculated by the Mie theory. Coating of anisotropic AgNPs, e.g., triangular nanoplates (AgTNPls) is more challenging as methods for silica coating of spherical AuNPs were found to be unsuitable for triangular nanoplates [113]. Silica coating of AgTNPls was achieved through a modified Stöber approach using TEOS as the alkoxide precursor and various primers: diaminopropane priming followed by reaction with TEOS (Figure 13A) that allowed tuning the thickness of the silica shell in the range 7 to 20 nm, or mercaptopropyltrialkoxysilane (either ethoxy or methoxy, MPTES or MPTMS, respectively) priming followed by silica deposition from sodium silicate (Figure 13B). This latter method using MPTES conveyed the highest stability towards salt, while retaining RI sensitivity comparable to that of the original uncoated particles (Figure 13C).

**Figure 14:**
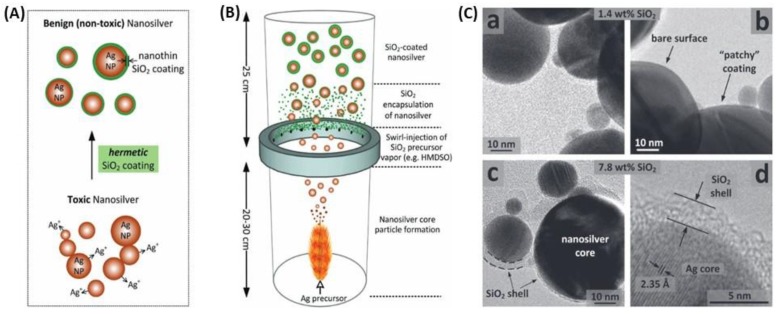
(**A**) Effect of a hermetic SiO_2_ coating on the flocculation and toxicity of nanosilver particles. (**B**) Illustration of the nanosilver encapsulation with a hermetic SiO_2_ coating using hexamethyldisiloxane as silica precursor in a flame aerosol reactor. (**C**) TEM images of the (a,b) 1.4 wt.% and (c,d) 7.8 wt.% SiO_2_-coated nanosilver. Adapted from [142]. Copyright (2010), WILEY-VCH Verlag GmbH & Co. KGaA, Weinheim.

The major interest of these particles is the wide tunability of the plasmonic energies which could have great attention in the development of biosensors [134]. The interest of silica coating was highlighted by Pratsinis and his group [142], who confirmed that silica coating prevented AgNPs agglomeration or flocculation then investigated of the plasmonic Ag@SiO_2_ NPs the toxicity against a model biological system (*Escherichia coli*) and concluded that it was blocked by coating nanosilver with a silica shell about 2 nm thick. The method used for silica coating is different from those described above as they used a flame aerosol method using hexamethyldisiloxane as silica precursor (Figure 14). To predict the LSPR biosensing performances, they measured the lambda shift upon the adsorption of bovine serum albumin (BSA). The response was improved but this improvement seems to arise from a better dispersion and therefore a higher amount of protein adsorbed, even if no experimental data was provided to confirm this hypothesis [142].

To conclude this part, the benefit from silica coating is well-established, in terms of chemical and colloidal stability and reduced toxicity. The protective silica shell has a limited effect on biosensing ability as long as the thickness of the layer is limited to few nanometers. Some aspects of silica coating, for instance the porosity, were not discussed herein, but are mentioned in the relevant review cited above. Beside these inputs from silica shells, there are no amplifying or synergetic effects in the plasmonic response of the Ag@SiO_2_ NPs, conversely to coating or mixing with gold, discussed in what follows.

## 4. Plasmonic Nanoparticles Based on Silver and Gold: Alloy, Core@Shell, Nanocages and Nanoshells

**Figure 15:**
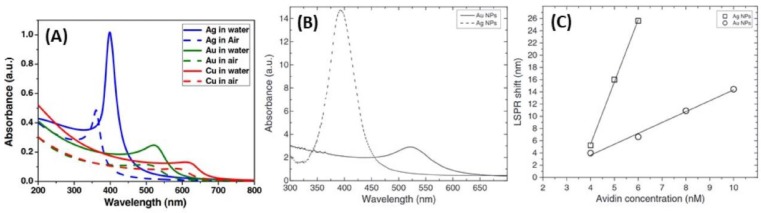
(**A**) Calculated extinction spectra of Ag, Au and Cu spherical NPs (20 nm) in different media. (**B**) Experimental extinction spectra of Ag and Au spherical NPs (10 nm) and (**C**) experimental response expressed as LSPR band shift of biocytin-coated Ag and Au spherical NPs (10 nm) in the presence of avidin. Adapted from [53,143]. Copyright (2014), Springer Science Business Media New York. Copyright (2012), Elsevier B.V. All rights reserved.

Compared to the two other plasmonic nanometals, i.e., gold and copper, silver nanoparticles have a higher theoretical refractive index sensitivity (RIS) (Figure 15) [53,143]. It has been shown that RIS increased from 153 to 265 nm/refractive index unit (RIU), 128 to 233 nm/RIU, and 117 to 212 nm/RIU, respectively, for AgNPs, AuNPs, and CuNPs with sizes from 5 to 50 nm. Spherical AgNPs exhibit a stronger LSPR absorption with a peak at 400 nm, which is from five to 10 times more intense than the gold one at 520 nm [53,143]. Despite this better sensitivity, observed both experimentally and theoretically, AgNPs have several drawbacks for biosensing; in addition to their poor stability and biocompatibility, they display less sensing reversibility due to light alteration, which makes their use in repeated cycles less reliable than that of AuNPs [80].

Many improvements have come about when AgNPs were combined to other metals and particularly to gold. Of course, the benefit from the previously discussed coating was effective with gold, but in addition, a synergy between these two plasmonic metals allowed for a better efficiency. This combination was mainly done by forming AgAu alloys or Ag@Au core@shell structures (Ag@AuNPs or Au@AgNPs). The main synthesis techniques and the resulting shapes and sizes are summarized in Table 3. In what follows, we will successively discuss AgAu alloys and Ag-Au core@shell structures synthesis. We will also cover the use of AgNPs as sacrificial templates to build gold nanocages (AuNC) or gold nanoshells (AuNS) for improvements of gold plasmonic biosensors.

### 4.1. Silver-Gold Alloy Nanoparticles

**Figure 16:**
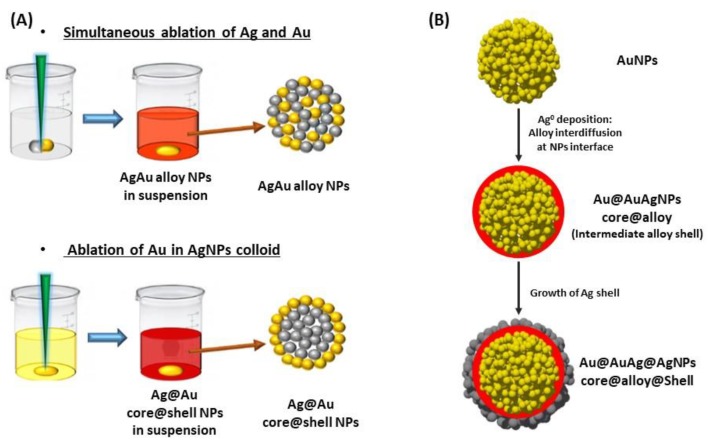
(**A**) Pulsed laser ablation in liquid: simultaneous ablation of Ag and Au to synthesize AgAuNPs and ablation of Au in AgNPs colloid to form core@shell structure (Au@AgNPs). Reproduced with permission from [145]. Copyright (2014,) Springer-Verlag Berlin Heidelberg. (**B**) Schematic illustration to form an intermediate AgAu alloy shell by interdiffusion at the NPs interface during hydrothermal treatment. Adapted from [148]. Copyright (2011), American Chemical Society.

Silver-gold alloy nanoparticles (AgAuNPs) are defined as a mixture of Ag and Au atoms, with no spatial distinction between the gold and silver parts. The chemical synthesis methods mainly consist in the co-reduction of AgNO_3_ and HAuCl_4_ with sodium citrate, which give spherical AgAuNPs [144,158]. The mole fraction of each metal in the alloy depends on the concentration of AgNO_3_ and HAuCl_4_ introduced in solution. In these conditions, small AgAuNPs (roughly 20 nm) can be synthesized [144]. Besides, simultaneous laser ablation of Ag and Au in colloidal suspension allows the synthesis of AgAuNPs, in the same range of size (Figure 16A) [145]. Another study demonstrated the AgAu alloy interdiffusion at the NPs interface, resulting in an intermediate alloy shell [148]. Indeed, a hydrothermal treatment is necessary during Ag^+^ reduction at the surface of AuNPs for Ag diffusion in Au in order to obtain AgAuNPs. This phenomenon is dependent on the temperature, such as the growth of the Ag shell layer until the final structure: core/alloy/shell.

AgAuNPs are also prepared by physical techniques, such as metal evaporation (electron beam), followed by thermal treatment which affords supported AgAuNPs on glass support [146,149]. Spherical or quasi-spherical AgAuNPs are obtained due to evaporation of Ag and Au layers in a vacuum chamber. Then, Au and Ag metallic atoms can be deposited on the glass substrate, following an island formation of AgAuNPs, because of the stronger interactions between Ag and Au atoms, than with the glass substrate [149]. The annealing post-treatment increases the crystallinity of AgAuNPs, but destroys completely pure AgNPs, previously synthesized in the same way [146]. In this technique, the AgAuNPs formation seems to be independent of the deposition order of the initial metallic layers on the glass substrate. Besides, in the case of the AuAgNPs elliptical formation with metal evaporation, the LSPR band shift is dependent on the shape, i.e., on the degree of sphericity of the AgAuNPs. AgAuNPs were also synthesized by UV laser radiation in the near-surface region of silicate glass [147]. Finally, nanosphere lithography allows the formation of very ordered arrays of silver-gold alloy nanoprisms (AgAuNPrs) on glass support. It has been shown that AgAuNPs are about four times more sensitive in RI than the equivalent supported spherical AgAuNPs with similar sizes and conditions. Moreover, the RIS of the AgAuNPs with x_Au_ = 0.5 is closer to pure AgNPs, i.e., very superior to pure AuNPs RIS [150].

**Figure 17:**
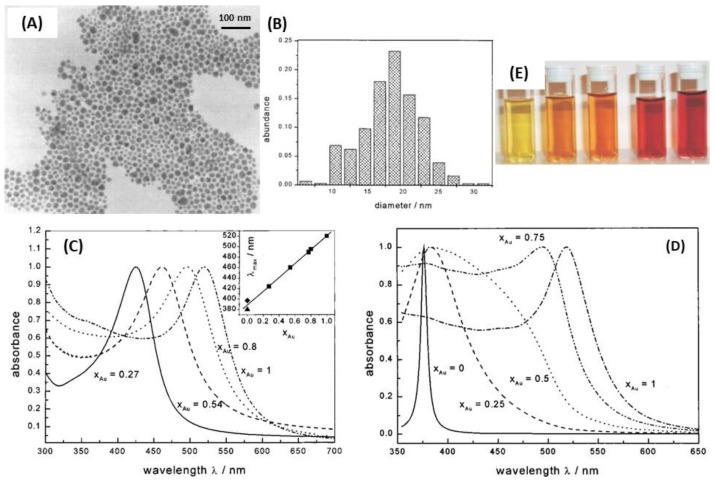
(**A**) TEM image and (**B**) size histogram of spherical AgAuNPs with Au mole fraction x_Au_ = 0.8: the average size is 18 nm. (**C**) Experimental and (**D**) calculated spectra regarding the LSPR shift of 18 nm diameter spherical AgAuNPs with varying Au molar fraction. (**E**) Colloidal suspensions of AuAgNPs with increasing Au concentration. Adapted from [92,144]. Copyright (1999), American Chemical Society. Copyright (2004), Elsevier B.V. All rights reserved.

AgAu alloys are more stable than gold-silver core@shell nanoparticles for the same size and shape [159]. The composition and the molar ratio between the two metals are important factors to be considered regarding their properties. Indeed, the plasmon peak for spherical AgNPs and AuNPs is around 400 and 520 nm, respectively, while the absorption of AgAuNPs can be tuned continuously from 400 to 520 nm *via* changing the alloy composition [34,160,161]. Qi et al. showed that the alloy NPs became less stable when Ag molar ratio increased conversely to core@shell NPs [159]. Hence, optical properties of alloys mainly depend on the ratio of one metal compared to the other, their size and shape. Indeed, AgAuNPs present only one peak, whose intensity and position in wavelength depend on the molar fraction of Au, x_Au_ [144,161]. In the case of spherical NPs of 18 nm average size, Link et al. demonstrated that the single LSPR peak of the AgAuNPs shifted from 400 nm (pure AgNPs) to 520 nm (pure AuNPs) according to the increasing gold molar fraction [144]. Indeed, it has theoretically predicted and experimentally observed that the LSPR band shift from 400 nm to 520 nm is linear, and proportional to the mole fraction of gold x_Au_. These results are shown in Figure 17. Moreover, theoretical simulations showed that the intensity of the peak decreases when the gold mole fraction increases [53,145].

The application of AgAuNPs to plasmonic biosensing is therefore based on the higher RIS of alloy NPs, compared to pure nanoparticles with equivalent size and shape. Indeed, BSA can be detected with AgAuNPs because a linearity between the LSPR red-shift and the concentration of BSA is observed for concentration between 0.1 and 100 ng/mL, which is better than pure AuNPs [146]. The sensitivity (i.e., the LSPR band shift) and the linearity can even be improved, with use of dopamine coated AgAuNPs.

### 4.2. Silver and Gold Core@Shell Nanoparticles

**Figure 18:**
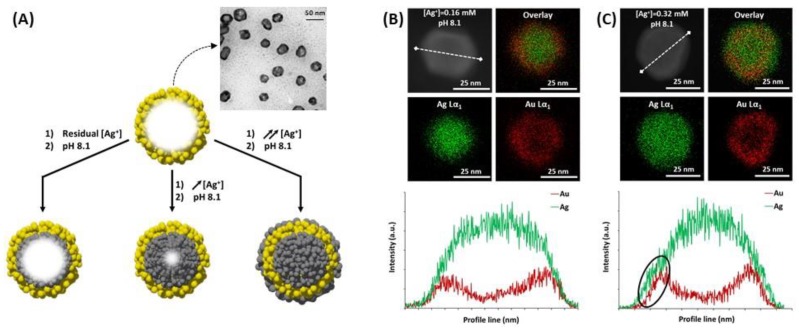
(**A**) Illustration of the reduction and growth process of Ag on the inner and outer surfaces of porous gold nanoshell (AuNS) with increasing amounts of Ag^+^ in the surrounding medium. STEM elemental mapping (Ag, Au, and overlay) of AuNS obtained after adding Ag^+^: (**B**) [Ag^+^] = 0.16 mM and the corresponding elemental profile along the white hatched line and (**C**) [Ag^+^] = 0.32 mM and the corresponding elemental profile along the white hatched line. The black ellipse in (**C**) highlights the reduction and growth of Ag at the external surface once the inner volume is completely filled. Adapted from [163]. Copyright (2019), American Chemical Society.

Silver and gold core@shell NPs are made of two spatially distinct layers, each containing a different element: a core, made of the first metal (Ag or Au), and a shell made of the second (Au or Ag, respectively). The core@shell notation places core first, thus, Ag@AuNPs refers to silver core coated by a gold shell and vice versa. The shell formation keeps the initial shape of the NPs, imposed by the metallic core, whatever isotropic or anisotropic [162]. The gold shell has essentially a protecting role on Ag core (Ag@AuNPs), to ensure the chemical stability of the previously synthesized AgNPs, and thus is often very thin. In this case, Au electrodeposition on Ag core is the main technique used with the possibility of successive voltammetric cycles to increase the thickness of the Au shell [151,152,157]. Other techniques exist, such as laser ablation of Au in a solution of Ag colloids, to generate the Ag core, on which the Au layer grows (Figure 16A) [53,145]. Here, the growth of the Au shell thickness is followed by UV-Vis spectroscopy, at different ablation times, according to the Mie theory, which makes the link between shell thickness and LSPR peak position. Chemical reduction of HAuCl_4_ at the AgNPls surface can also lead to very thin Au shells, by adding very slowly the gold solution [153]. The main problem in these techniques is to avoid galvanic replacement of Ag by Au, which would destroy at least partially the Ag core. Indeed, Ag is a more reductive metal than Au. Recently, our group introduced an original pathway to form Ag@AuNPs from hollow gold nanoshell (AuNS) [163]. Porous AuNS were prepared by galvanic replacement starting from AgNPs generating Ag^+^ ions in the process (Section 4.3 see below). Increase of pH in the presence of these AuNS triggers the reduction of Ag^+^ that preferentially occurs at the inner walls of AuNS. The reaction initially relies on the presence of residual Ag^+^ inside the AuNS as well as in the surrounding solution, and it proceeds upon external addition of Ag^+^ until a solid Ag core is formed inside the AuNS to form Ag@AuNPs (Figure 18). Then, subsequent reduction of Ag^+^ occurs on the external surface of the solidified AuNS (Ag@Au@AgNPs). Controlling the Ag content in AuNS allows tuning the LSPR band position at the desired wavelength for biosensing applications.

Regarding the synthesis of Au@AgNPs, the chemical reduction of AgNO_3_ at the AuNPs surface is the main technique used. This requires a reducing agent, which is very often citrate/ascorbate, to form spherical and rod-shaped core@shell structures by Ag chemical deposition on Au core [154,155,156,161,162]. Besides, a very thin Au layer can be electrodeposited after the Au@AgNPs synthesis, making a peculiar structure, called Au@Ag@AuNPs [157]. Liz-Marzán et al. also realized successive reduction of AgNO_3_ and HAuCl_4_ in the presence of AA and CTAB on preformed Au seeds to obtain multilayer bimetallic nanoparticles (Au@Ag, Au@Ag@Au, and Au@Ag@Au@Ag NPs) [164]. According to the Mie theory, for isotropic nanoparticles due to the hybridization between two different plasmonic nanoparticles, the LSPR spectrum should display two peaks, one coming from the core@shell interface between the two metals, and the other one coming from the surface of the shell [53,165]. The position of the former peak mainly depends on the core metal, while the position of the latter one mainly depends on the shell metal, but also on the thickness of the shell layer. As described previously, pure AgNPs have an LSPR extinction peak around 400 nm, and more intense than pure AuNPs. It can be expected that the optimal LSPR properties should occur for very thin Au shells regarding Ag@AuNPs, whereas the Ag shell can be thicker for Au@Ag NPs. Indeed, Zhu et al. demonstrated that the peak of Ag@Au nanowires red-shifted and the intensity decreased when the Au shell thickness increased. In addition, the shell peak is almost inexistent for low Au shell thicknesses. While the peak blue-shifted when the thickness of Ag shell was increased, and the shell peak was more intense for Au@Ag nanowires [166].

**Figure 19:**
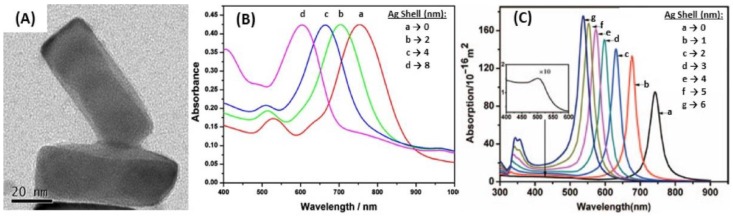
(**A**) TEM image of Au@Ag nanorods with 60 × 20 nm dimensions for the Au core and 4 nm thickness for the Ag shell and (**B**) variation of the extinction spectra of Au@Ag nanorods with varying Ag shell thickness (0–8 nm) on the 60 × 20 nm Au core. (**C**) The variations in the calculated LSPR spectra of Au@AgNRs with varying Ag shell thickness (0–6 nm) as well as the zoom on the spectrum allowed seeing the peak corresponding to the Au-Ag interface transversal resonance. Adapted from [154,156]. Copyright (2014), American Scientific Publishers. Copyright (2014), Springer-Verlag Wien.

Besides, the anisotropic core@shell NPs such as NRs, for which the synthesis was widely described [161,162], the peaks coming from the core@shell interface and the shell surface were enhanced because of the presence of various favored directions, but not all the resulting peaks were always observable. Indeed, four peaks should be observed for Au@AgNRs but only three were actually observed because the two initial peaks were split due to the presence of two favored directions [155]. Moreover, only two remained observable when Ag shell thickness increased. These two peaks corresponded to the longitudinal resonances of the Ag external shell for the shorter wavelength, and Au-Ag interface for the higher wavelength, as in the case of spherical Au@AgNPs [155,167]. The most intense peak observed corresponding to the longitudinal resonance of the Au-Ag interface blue-shifted when the Ag shell thickness increased [156,168]. However, no linearity was observed for Au@AgNRs between the LSPR shift and the Ag shell thickness from 740 nm (Ag shell: 0 nm) to 507 nm (Ag shell: 6 nm), based on the calculated spectra [154]. Figure 19 shows the results in terms of LSPR band position for different Ag shell thickness.

**Figure 20:**
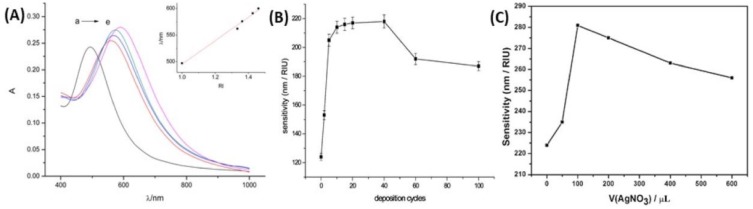
(**A**) Localized surface plasmon resonance (LSPR) band shifts for Ag@Au hemispherical nanoplates (40 nm radius) supported on ITO glass with increasing RI media (a: air, b: water, c: ethanol, d: cyclohexane, e: carbon tetrachloride), and the linear relation between shift and RI (inset). (**B**) Evolution of the RIS with the number of Au shell electrodeposition cycles on the Ag core for the previous Ag@Au nanoplates. (**C**) The evolution of the RIS with the concentration of AgNO_3_ for the deposition of the Ag shell on the 20 × 60 nm Au core in the case of Au@AgNRs (TEM image was previously shown in Figure 19A). Adapted from [152,156]. Copyright (2013), American Chemical Society. Copyright (2014), Springer-Verlag Wien.

Several studies pointed out the interesting properties of the core@shell structures based on Ag and Au for LSPR biosensing, because of their high RIS [155]. The LSPR band red-shift is observed when the surrounding RI increases for both Ag@Au and Au@Ag structures [152,156]. Moreover, considering one LSPR peak, the shift in wavelength is proportional to the RI, which is useful for detection of biomolecules in solution [157]. The RIS of core@shell NPs is also dependent on the size and the shape: anisotropic core@shell nanoparticles (NRs, TNPls, etc.) are more sensitive [38] than alloy NPs [169] or anisotropic and isotropic pure NPs [156]. As it has already been described, in the extinction spectrum, there is both an influence of the core/shell interface (depending on the two metals, as in alloys) and shell thickness (depending on the shell metal, as in pure NPs). Additionally, it is possible to deposit a dielectric layer at the surface of Au@Ag, to improve the whole stability as Ag is less stable than Au [170]. In this case, it has been shown that the LSPR sensitivity is not lost, but it can even be raised. Indeed, the LSPR band position increases when the permittivity of the layer is higher than the permittivity of the surrounding medium. The interesting LSPR properties of the core are kept if the protecting layer, which improves stability, is very thin, compared to the core size [151]. Moreover, Dong and his co-workers showed that a certain number of cycles is required for the best efficiency, both for the homogeneity in size and shape and for the RIS in the case of successive Au deposition on Ag core with voltammetric cycles [152,157]. A similar observation was realized for the chemical reduction of Ag on Au, for which a certain quantity of AgNO_3_ is required for the best sensitivity in RI [156]. Figure 20 shows the linearity between LSPR peak shift and RI, as well as the higher sensitivity of Au@AgNRs compared to Au@AgNPls. A study has also showed Au@Ag core–shell nanorods have better SERS responses, compared AuNRs [154]. Indeed, the SERS intensities increased with the increase of the Ag shell thickness, which demonstrates that the composition and morphology of NPs play key roles on the SERS signals.

### 4.3. Destructive Use of Silver Nanoparticles with Gold

**Figure 21:**
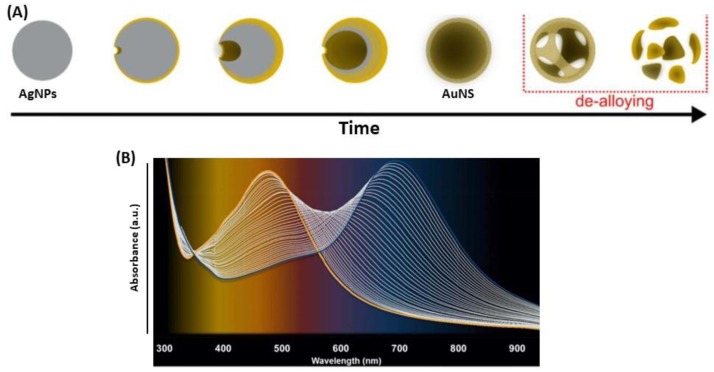
(**A**) The structural evolution of AuAg nanostructures during the galvanic replacement reaction upon addition of HAuCl_4_ and (**B**) absorption spectra evolution as a function of time of AgNPs titrated with increasing volumes of HAuCl_4_ to form AuNS: the LSPR band gradually shifts through the whole visible spectrum toward NIR wavelengths. Adapted from [175]. Copyright (2018), American Chemical Society.

AgNPs can be used as sacrificial template in the destructive way for the synthesis of gold nanobowls [171], gold nanocages (AuNC) [172,173] or nanoshells (AuNS) (Figure 21A) [79,163,174,175]. Indeed, these structures are synthesized by a galvanic replacement reaction, where the metallic salt with higher reduction potential is added to a suspension containing a metal nanoparticle with lower reduction potential, as the following reaction [174,176]:

Au^3+^_(aq)_ + 3 Ag_(NPs)_ → 3 Ag^+^ + Au_(NC/NS)_

Specifically, the standard reduction potential of Au^3+^/Au redox pair is 0.99 V vs. the standard hydrogen electrode (SHE), whereas the standard reduction potential of Ag^+^/Ag is 0.80 V vs. SHE [163]. The difference in reduction potential causes Au to be deposited on the Ag template upon release of Ag^+^ into the solution. This method allows designing the shape of the NC or NS as the complementary shape of AgNPs. The nanoshell geometry is ideal for tuning and optimizing the near-field response for SERS on substrates and optical resonance properties of biosensors (Figure 21B) [76,79,174,175]. Tuning the LSPR band of nanoshells into the NIR spectral range leads to a variety of bioapplications.

Regarding the AuNC, the synthesis is done in two steps. The first one is the AgNPs synthesis by electrodeposition on a glass support. Then, the second step consists in the galvanic replacement of Ag by Au, at the AgNPs surface, which can be followed by UV-Vis, using the variation of intensity of the LSPR Au (increasing) and Ag (decreasing) bands, and by cyclic voltammetry. For complete removal of Ag, i.e., to complete gold-silver dealloying, it is necessary to use oxidizing agents such as nitric acid (HNO_3_) or hydrogen peroxide (H_2_O_2_) [173]. Another way to synthesize AuNC is to start from Ag disks deposited on a glass with colloidal lithography, followed by galvanic replacement of Ag by Au to create peculiar AuNC, which are AuAg nanobowls [171].

## 5. Selected Applications of Ag and AgAu-Based Plasmonic Nanoparticles in Optical Biosensing

Few studies have reported the use of Ag and AgAu-based plasmonic nanoparticles for biosensor applications. In this last part, selected examples of the use of Ag and Ag-Au nanoparticles in the development of plasmonic biosensors based on biomolecules recognition will be detailed.

### 5.1. RI-Based LSPR Biosensors

**Figure 22:**
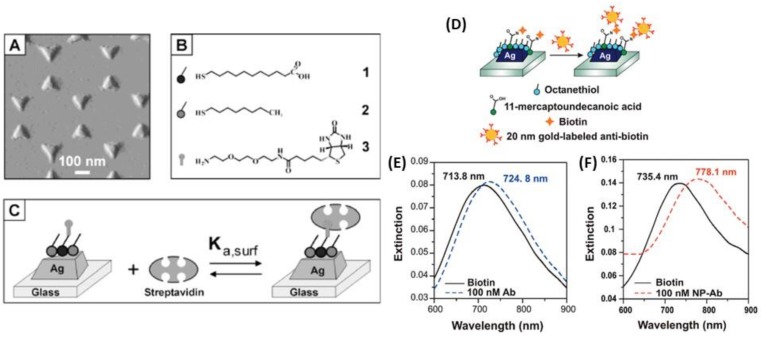
Silver triangular nanoparticles fabricated by NSL on a glass substrate. (**A**) Tapping mode AFM image of the Ag triangular NPs. (**B**) Surface chemistry of the Ag nanobiosensor. A mixed monolayer of (1) 11-MUA and (2) 1-OT is formed on the exposed surfaces of the AgNPs followed by the covalent linking of (3) biotin to the carboxyl groups of (1) 11-MUA. Schematic illustration of (**C**) streptavidin binding to a biotinylated Ag nanobiosensor and (**D**) biotin covalently linked to the Ag nanobiosensor surface while antibiotin-labeled AuNPs are subsequently exposed to the surface. LSPR spectra (**E**) before (solid black) and after (dashed blue) binding of native antibiotin and (**F**) before (solid black) and after (dashed red) binding of antibiotin-labeled NPs. Adapted from [57,177]. Copyright (2002, 2011), American Chemical Society.

Although AgNPs have been used less extensively than AuNPs in the development of biosensors, very interesting works have been published in LSPR optical biosensing. Indeed, a study showed the use of AgNPs exhibited better results for RI-based LSPR biosensing compared to AuNPs, as discussed previously in the manuscript. The LSPR band shift resulting from the addition of biocytin-coated metallic nanospheres by addition of avidin was approx. 5 times higher for AgNPs than for AuNPs, 1.78 nm/nM vs. 10.18 nm/nM for AuNPs and AgNPs, respectively (Figure 15C) [143]. Another study investigated the development of Ag triangular plasmonic NPs on glass substrate, fabricated by NSL, to lead to sensitive and selective nanoscale affinity biosensors for the streptavidin-biotin couple [57]; the limit of detection (LOD) for these LSPR biosensors was found to be in the low-picomolar to high-femtomolar region (Figure 22A–C). A method to amplify the wavelength shift, previously observed, from LSPR bioassays was optimized using Au nanoparticle-labeled antibiotin antibodies. After binding an antigen to the antibody-conjugated Ag nanotriangles, a secondary antibody attached to AuNPs was added. The resulting plasmonic coupling between the Ag nanotriangles and the Au colloids reduced the LOD by three orders of magnitude for more sensitive detection (Figure 22D–F) [177].

**Figure 23:**
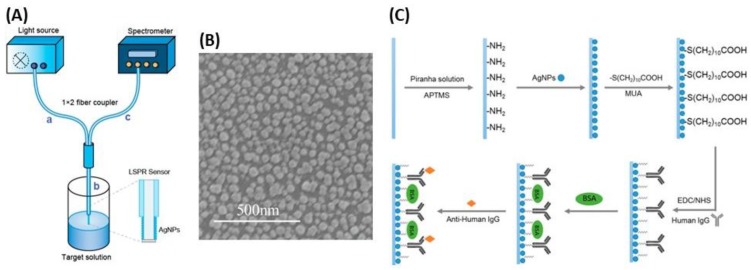
(**A**) Schematic illustration of the experimental set-up used for the LSPR optical fiber sensor. (**B**) SEM image of immobilized AgNPs on optical fiber surface. (**C**) Illustration of the employed strategy for the development of LSPR optical fiber biosensors based on AgNPs. Adapted from [179].

To enhance the sensitivity of the LSPR optical sensor, a new and recent approach used by depositing NPs on an optical fiber. The principle of LSPR optical fiber sensors is also based on the plasmon resonance of metal NPs, but coated on optical fiber surfaces, that are more sensitive to changes in the surrounding medium [178,179]. The label-free and real time detection proposed by this technology is a valuable asset compared to classical techniques. However, there are few studies regarding the development of LSPR optical fiber sensors with AgNPs, although nanosilver films have been proven to be much more sensitive to surrounding medium changes than other metal films [180]. Among these studies, Chen et al. proposed a stable and sensitive reflective LSPR optical fiber sensor based on AgNPs to optimize the fabrication process, including two key parameters (the sensing length and the coating time) [179]. The surface of AgNPs deposited on the optical fibers was then functionalized with an antibody and the antigen-antibody binding process was optically monitored by measuring the wavelength shift in real time (Figure 23). This technique gave a RIS of 387 nm/RIU, which is much higher than that reported for colloidal suspension of AgNPs. Another study found a RIS of 67.6 nm/RIU by photodepositing of AgNPs on the optical fiber end [178]. The sensor response is such that the LSPR peak wavelength is linearly shifted to longer wavelength as the RI is increased.

Besides, Patora and Astilean developed LSPR biosensors based on chitosan-coated AgNPs to devise a multi responsive plasmonic sensor [91]. They exploited the anisotropic AgNPs as LSPR chemosensors and *p*-ATP as the target. They showed a gear of plasmon resonance peak, which allows a greater shift toward higher wavelengths. In this same study, chitosan-coated NPs were also used as LSPR sensors for monitoring trace amount of adenine by shifts of LSPR bands proving the binding between the particles and adenine, showing that, the chitosan coated AgNPs make sensitive LSPR sensors and good SERS substrates.

**Figure 24:**
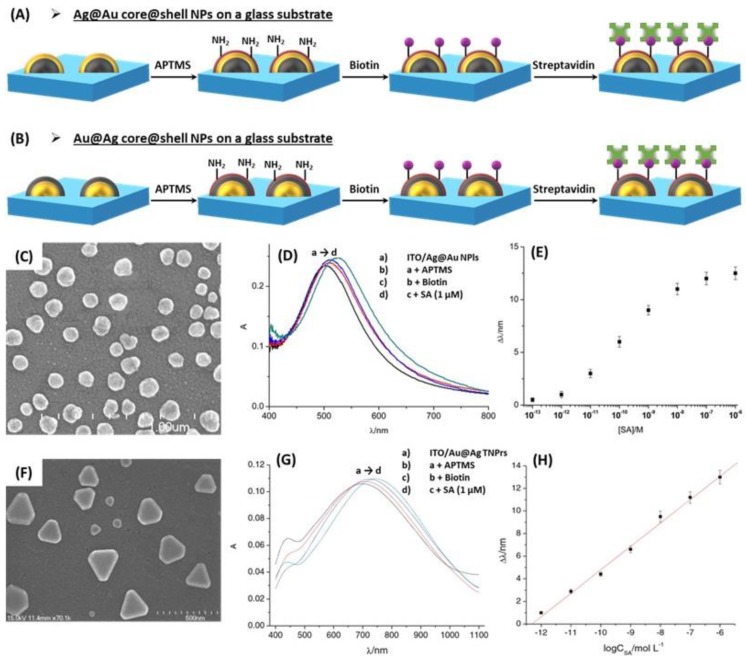
(**A**,**B**) Schematic illustration showing the preparation of glass-supported core@shell NPs for SA biosensing. (**C**) SEM image of Ag@Au hemispherical nanoplates supported on ITO glass. (**D**) LSPR peak (500 nm) was shifted upon successive treatments with APTMS, biotin and SA. (**E**) Relationship between the LSPR band shift and SA concentration for Ag@Au NPls. (**F**) SEM image of Au@AgTNPls supported on ITO glass. (**G**) LSPR peak (700 nm) shifted upon successive treatment with APTMS, biotin and SA. (**H**) Linear relationship between the LSPR band shift and SA concentration. Adapted from [152,157]. Copyright (2013), American Chemical Society. Copyright (2013), Springer Science Business Media New York.

The applications of core@shell NPs combining Ag and Au for RI-based LSPR biosensors are also presented. A study showed that the optical properties of the Au@Ag core@shell NPs were similar to those of pure AgNPs for a given sizes, which was confirmed by means of Mie extinction calculations, while the SERS properties of Au@AgNPs exhibited a higher efficiency than AgNPs under near-infrared excitation [181]. Moreover, the results of three studies focusing on the detection of streptavidin (SA) in solution are briefly discussed below, as a model of optical biosensor, based on the RIS of glass-supported core@shell NPs, and using the receptor-analyte recognition (Figure 24A,B). Biotin, which interacts strongly with the SA target molecule, is fixed on the external shell of the core@shell NPs, which was previously amine-functionalized with 3-aminopropyltrimethoxysilane (APTMS). Two studies used Au@Ag structures, and one Ag@Au structure. Indeed, the SA detection in solution was proven with the use of Au@AgNRs [156], Au@Ag triangular nanoprisms (TNPrs) and Au@Ag@AuTNPrs [157] as well as Ag@Au hemispherical NPls [152]. The results are very comparable: successive shifts in the LSPR peak are observed upon successive additions of APTMS, biotin and SA, in correlation with the induced changes in RI. In addition, linearity is always observed between the LSPR peak shift and SA concentration, as a result of change of local RI. Figure 24C–H shows that the results for SA detection are very similar for Ag@Au and Au@Ag structures, except that the position of the main LSPR peak is red-shifted with Au@Ag core@shell. The Au@AgNRs being more sensitive to RI change, are also more sensitive with respect to SA detection [156] than NPls. Besides, the Au@AgTNPrs have been further coated with a very thin layer of Au. The resulting (Au@Ag@AuTNPrs) keep the initial sensitivity properties of the Au@AgTNPrs, and the linearity between LSPR peak shift and SA concentration [157]. Regarding Ag@Au hemispherical NPls, a complementary study on the biodetection of immunoglobulin G with anti-immunoglobulin G bound Ag@Au NPs, showed similar results to those with SA [152].

Finally, the fabrication of substrate-bound AuAg nanobowls arrays synthesized through the galvanic replacement of silver disk arrays is used for size-selective LSPR biosensors. This sensor should prove useful in both size determination and differentiation of large analytes in biological solutions, such as viruses, fungi, and bacterial cells. In these devices, both the LSPR and the SERS signals are enhanced, and the LSPR peak is red-shifted, when the target analyte is small enough to penetrate inside AuAg nanobowls. Otherwise, the previous described effects on LSPR and SERS are not observed [171]. Therefore, this concept was applied towards the detection of a 95 nm H1N1 virus, where the larger diameter nanobowls showed an increased plasmonic response upon addition of the virus.

### 5.2. Ag and Mixed AgAu Nanoparticle-Based Colorimetric Biosensors

This approach received considerable attention in the analytical field for naked-eye detection due to its simplicity and low cost, it does not require any expensive or complex instrumentation. Due to these inherent optical properties, colloidal suspensions of AgNPs and mixed Ag and Au NPs have high extinction coefficients and different colors in the visible region of the spectrum when they are dispersed in comparison with when they are aggregated.

#### 5.2.1. Ag Nanoparticles Aggregation-Based Colorimetric Assays

**Figure 25:**
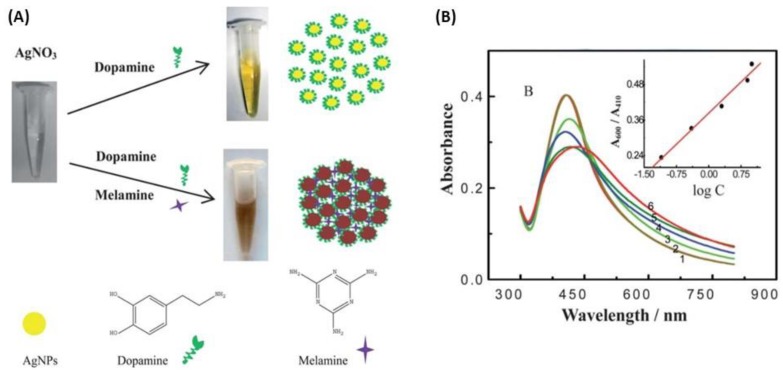
(**A**) Principle of nanoparticle aggregation based-colorimetric assay for the melamine detection with dopamine-modified AgNPs. (**B**) UV-Vis spectra of dopamine-stabilized AgNPs suspensions with different melamine concentrations: (1) 0 mM, (2) 0.08 mM, (3) 0.4 mM, (4) 2 mM, (5) 8 mM and (6) 10 mM. Adapted from [184]. Copyright (2011), Royal Society of Chemistry.

In literature, the development of nanoparticle aggregation based-colorimetric assays has been reported. The optical plasmon properties of AgNPs depend strongly on the interparticle distance between pairs of NPs, small or large aggregates of AgNPs as compared to individual and well-spaced NPs. A decrease in the interparticle distance leads to a strong overlap between the plasmon fields of the nearby particles, causing a redshift in the LSPR band with an increase in intensity and an easily observable change in color solution. Indeed, the analytical performance with high sensitivities because of the strong LSPR and excellent selectivity driven by the interaction between analyte-NPs and its surroundings involving mainly electrostatic and hydrogen bond interactions as well as donor–acceptor chemical reactions. Therefore, a well-designed chemical interaction could lead to a change of color for naked-eye detection of the target analyte [182]. AgNPs-based colorimetric assays have been investigated for melamine detection [183,184]. Han et al. used *p*-nitroaniline (*p*-NA)-modified AgNPs, as a sensitive, selective and simple colorimetric assay, resulting in a color change from yellow to blue in the presence of melamine [183]. This optical method was highly reproducible and concentrations as low as 0.1 ppm of melamine in infant formula can be visualized by the naked-eye. The same strategy was proposed by Ma et al. with dopamine-stabilized AgNPs to detect visually the melamine [184]. Indeed, the color change of the suspension turned from yellow to brown (Figure 25). The results showed the concentrations of detectable melamine were in the range of 10 ppb to 1.26 ppm. AgNPs functionalization and the analysis of melamine can occur in one-step since *p*-NA or dopamine acts as a reducer and stabilizer of AgNPs and as a linker of melamine molecules. Other biomolecules has also been detected by a colorimetric sensor highly selective such as tryptophan [185] or histidine [186] thanks to organic coating on AgNPs: 4.4-bipyridine (4-DPD)- and *p*-sulfonatocalixarene (*p*-SC4)-modified AgNPs, respectively. In the first case, tryptophan interacts with the pyridine ring of 4-DPD *via* π-π interactions, and meanwhile carboxyl acid of tryptophan can also form hydrogen bonds with pyridine, which results in 4-DPD-functionalized AgNPs aggregation and the color change from yellow to red. The LOD was 20 µM for the tryptophan colorimetric detection [185]. In the second case, the aggregation process is due to *p*-SC4, which possesses an electron-rich cyclic cavity, being able to fit imidazole and the side chain of histidine *via* host–guest electrostatic and cation-π interactions. The color change turned from yellow to red and the LOD of 5 µM was obtained [186]. Besides, a derivative of calixarene has also been employed for colorimetric detection of pesticides in water [187].

#### 5.2.2. Mixed AgAu Nanoparticles-Based Colorimetric Assays

**Figure 26:**
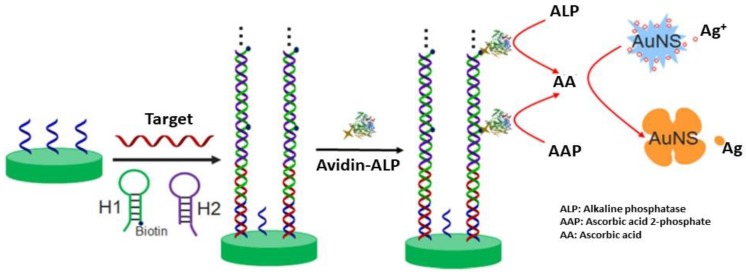
DNA detection by naked-eye readout with the silver reduction on gold nanostars [188]. Copyright (2015), Elsevier B.V. All rights reserved.

A colorimetric biosensor with naked-eye detection was designed in which target DNA was indirectly detected through reduction of Ag^+^ to AgNPs at the surface of gold nanostars coated by capture probe (Figure 26) [188]. In the presence of target DNA, biotin-labeled complementary oligonucleotide H1, oligonucleotide H2 and avidin-alkaline phosphatase conjugate, ascorbic acid 2-phosphate is converted into AA, which acts as reducer. The resulting observation is a blue-shift of the LSPR peak spectrum, due to the formation of Ag shell on gold nanostars. This technique showed a detection range from 10 fM to 50 pM DNA with a detection limit of 2.6 fM.

**Figure 27:**
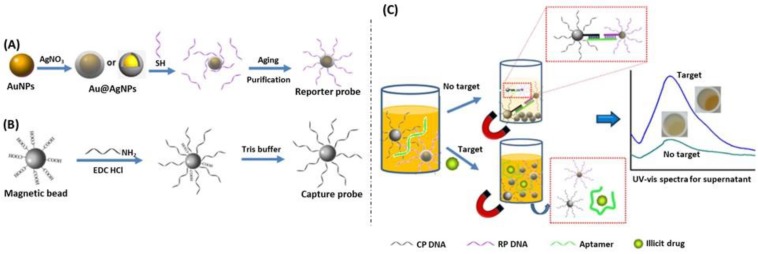
Schematic illustration of the preparation of the (**A**) reporter probe and (**B**) capture probe as well as the principle of the colorimetric detection of illicit drug based on non-aggregation Au@Ag core@shell NPs. Reproduced with permission from [189]. Copyright (2017), Published by Elsevier B.V.

A more recent technique based on non-aggregated Au@Ag core@shell NPs was developed by Mao et al. to detect drugs, such as cocaine, using the coloration of the solution containing NPs [189]. For this purpose, they have used Au@Ag nanoparticles coated with a DNA aptamer specific of cocaine and magnetic beads coated with a DNA sequence, partially complementary to the aptamer sequence allowing cross-linking of Au@Ag nanoparticles and magnetic beads. In the presence of a magnetic field, the nanoparticles leave the suspension with the magnetic beads, and the solution is slightly colored. When cocaine is added to the solution, it interacts with the aptamer, destroying the link between nanoparticles and magnetic beads, allowing the coloration of the solution by the nanoparticles (Figure 27). This study shows the new major improvements in the silver plasmonic biosensing.

### 5.3. Metal-Enhanced Fluorescence (MEF)-Based Biosensors

**Figure 28:**
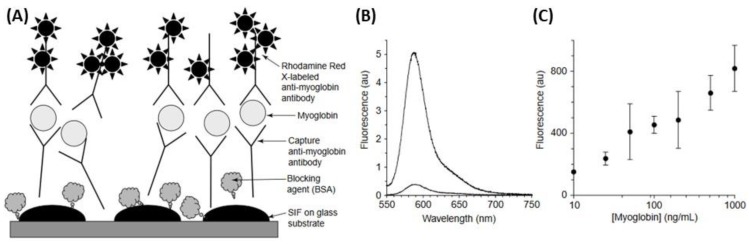
(**A**) Illustration of a metal-enhanced sandwich immunoassay on silver island films (SIFs). Fluorescence emission of the Rhodamine Red-X-labeled anti-myoglobin antibody attached to the surface-immobilized myoglobin (**B**) for a given myoglobin concentration (100 ng/mL) on SIFs and on glass, and (**C**) at different myoglobin concentrations on SIFs. Adapted from [191]. Copyright (2005), Elsevier Ltd. All rights reserved.

Although fluorescence is a highly sensitive technique, where single molecules can readily be recognized, the detection of a fluorophore is usually limited by its quantum yield, auto-fluorescence of the samples and/or the photo-stability of the fluorophores. However, the use of metallic nanostructures such as silver allows modifying favorably the spectral properties of fluorophores and reducing some of these fluorophore disadvantages for metal-enhanced fluorescence (MEF) [190,191]. An interesting study has reported the use of a relatively facile deposition of AgNPs onto glass slides (i.e., silver island films, SIFs). This sensor was used for the development of an enhanced detection limit sandwich-format immunoassay for the cardiac marker myoglobin (Figure 28A) [191]. Indeed, the SIFs and glass surfaces were coated with anti-myoglobin antibodies, and then incubated with fluorophore-labeled anti-myoglobin antibodies. This approach of metal-enhanced planar immunoassay showed a 10–15-fold increase in fluorescence emission observed on the SIFs compared to that naked substrate (Figure 28B) and the results demonstrated the myoglobin concentrations were detected in the 10–1000 ng/mL range (Figure 28C).

AgNPs functionalized with various thiolates have been also explored for MEF-based biosensors [191]. Indeed, DNA hybridization assays using metal-enhanced fluorescence (MEF) were investigated with thiolated oligonucleotides, which were bound to AgNPs on a glass substrate. This approach suggested the use of AgNPs improved the sensitivity of DNA detection with an increase in the number of detected photons per fluorophore molecule by a factor of 10-fold or more [192]. In addition, another study has also used thiol-organic monolayer-protected AgNPs, which were displaced by oligonucleotides through ligand exchanges, and a fluorophore-labeled complementary oligonucleotide were employed for DNA hybridization. The results showed a possible approach to DNA detection with a surface-enhanced emission after hybridization in the presence of AgNPs based on the aggregation of AgNPs bound by fluorophore-labeled oligonucleotides [193]. Finally, Ag@SiO_2_ NPs have been also exploited as transducers of DNA hybridization [141], or to achieve MEF-based biosensor [194,195]. In the latter case, 3- to 5-fold enhanced fluorescence signals can be obtained from SiO_2_-coated AgNPs colloids labeled with cyanine and by their aggregation in suspension. This inert coating reduces the close proximity quenching by noble metals, as well as provides for a wide variety of chemistries for biomolecule attachment. AgNPs@SiO_2_ can thus become a solution-based enhanced-fluorescence sensing platform [190,191,194].

### 5.4. Optical Biosensors Based on the Oxidation of Ag

**Figure 29:**
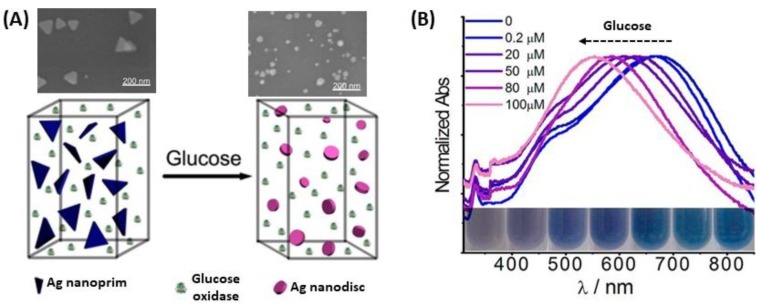
(**A**) Illustration of the strategy to modify the AgNPs shape from nanoprisms to nanodiscs through Ag oxidation for colorimetric sensing of glucose. SEM images of the Ag nanoprisms before and after incubation with glucose oxidase and glucose (100 μM) for 60 min are also showed. (**B**) Absorption spectra of the Ag nanoprisms after glucose incubation in various concentrations for 40 min with photographs of the corresponding suspensions. Adapted from [196]. Copyright (2013), American Chemical Society.

The oxidation of silver from AgNPs and mixed AgAu NPs, even in destructive way, can be exploited for optical biosensors. Indeed, a work has used the highly reactive properties of H_2_O_2_ to modify the nanoparticle shape for improved detection by colorimetric visualization. Xia et al. used the enzyme glucose oxidase mixed in solution with Ag nanoprisms to catalyze the reaction between glucose and oxygen to form H_2_O_2_ and gluconic acid. As the reactive H_2_O_2_ etched the tips of the nanoprisms, drastic shape and color changes were observed in the LSPR spectrum, resulting in a detection range from 0.2 µM to 100 µM in diluted serum (Figure 29) [196].

**Figure 30:**
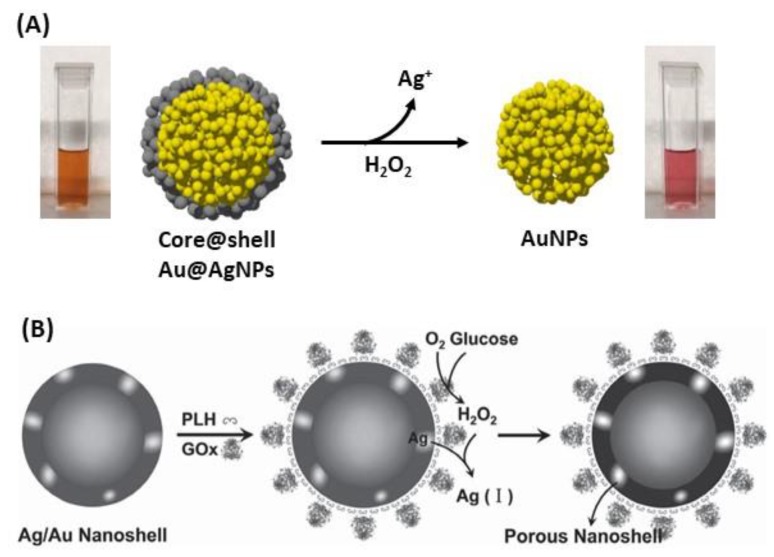
(**A**) Illustration showing the oxidation of Ag shell on Au@Ag core@shell NPs to Ag^+^ in the presence of H_2_O_2_ produced by an enzymatic reaction. (**B**) Schematic illustration showing the glucose sensing mechanism with Ag/Au nanoshells. Adapted from [198]. Copyright (2012) WILEY-VCH Verlag GmbH & Co. KGaA, Weinheim.

Furthermore, the destructive use of AgNPs is described by Ag oxidation for the synthesis of gold nanocages (AuNC) as well as hollow gold nanoshells (AuNS). First, the dissolution of the Ag part of the NPs is followed by the changes in the initial UV-Vis spectrum, either the shift of the peaks, or the changes in absorbance allow quantifying the presence of the biomolecule [197]. Several studies also demonstrated that glucose can be detected by this technique, using gold-silver nanoshells (AuAgNS) or Au@AgNPs [198,199]. In both cases, Ag is oxidized to Ag^+^ because of H_2_O_2_ produced (Figure 30A) from glucose oxidation to gluconic acid in the presence of dioxygen (O_2_) and glucose oxidase. This strategy is based on the quantification of the amount of metallic Ag oxidized, then the quantity of H_2_O_2_ is determined as well as the quantity of glucose. In 2012, a study on Ag oxidation in presence of glucose with dioxygen and glucose oxidase in AuAgNS showed that the LSPR peak is red-shifted with increasing glucose concentration (Figure 30B) [198]. There is linearity between the wavelength shift and the glucose concentration at very low concentrations (down to 20 µM). In another study, the same oxidation process of Ag in presence of glucose with O_2_ was observed in Au@Ag core@shell structures. The LSPR band intensity, at fixed wavelength was correlated to glucose concentration. In this case, a linearity was observed between absorbance and glucose concentration for higher glucose concentrations (down to 400 µM), and a similar observation has been made for cholesterol detection, using the same method of Ag oxidation [199].

## 6. Conclusions

The interest of silver nanoparticles as highly sensitive materials for plasmonic biosensors design is well-established. Indeed, although AgNPs are less chemically stable and less biocompatible compared to AuNPs, they provide more sensitive plasmonic biosensors owing to their LSPR features. The AgNPs synthesis is now well mastered and well described allowing the fabrication of differently shaped particles from the simplest to special uncommon shapes thanks to the large range of synthesis techniques now available and described in this manuscript for a conceptual opportunity in biosensing. This is a real advantage to explore many more properties. The coating, either organic or inorganic, overcomes the issues of stability and toxicity raised above and allows for the use of the resulting core@shell nanoparticles in plasmonic biosensing. Finally, the use of gold with silver nanoparticles, in alloy and core@shell structures, also provides a protective shell but in addition, enhances the plasmonic response of the resulting colloids.

Most of these findings are recent, and this may explain the few biosensing applications based on AgNPs compared to AuNPs to date. We expect growing interest to the application of these nano-objects in biosensing field, thanks to their higher RIS that allows for a better sensitivity when the strategies are based on the shift of the LSPR band. They are also very promising in naked-eye detection strategies, where multiple scenarios can be envisioned including aggregation, visible lambda shift, or even destruction of a silver shell on a gold core. In this review, the majority of selected applications of Ag and mixed AgAu nanoparticles-based plasmonic biosensors represents only trivial biosensing schemes to emphasize the merit of Ag-related NPs and provide the future prospects silver-based plasmonic nanoparticles in biosensing. In such an application, the expectations for an on-site biosensor, i.e., sensitive, reliable, fast, and user-friendly, are completely fulfilled.

## Figures and Tables

**Table 1:** Summary of the main strategies employed to synthesize silver nanoparticles (AgNPs) along with the corresponding shape and size, as well as their respective applications.

Shapes	Sizes (nm)	Synthesis Methods	Applications	Ref.
Spheres	10–100	Chemical reduction	Plasmonic and sensing; catalysis; antimicrobial	[55,56]
Triangles	Width: 100 Height: 50	Chemical reduction; nanosphere lithography (NSL)	Plasmonic and sensing; analytical devices (SERS); photovoltaics; molecular detection (Alzheimer disease)	[56,57,58,59]
Nanocubes	20–45	Polyol process	Cysteine sensing by plasmons of silver nanocubes; analytical devices (SERS)	[54,60,61]
Nanowires	Length: 60	Polyol process	Plasmonic and molecule sensing; provide conductive coatings (transparent conductors and flexible electronics)	[54,62,63]
Nanorods	Length: 250–300	Photochemical; thermal; oxidation reduction growth (ORG)	Plasmonic and sensing; analytical devices (SERS)	[54,64]
Nanobars	Length: 100	Polyol process	Plasmonic and sensing; analytical devices (SERS)	[54,56]
Pyramides	Edge length: 50–200	Chemical reduction	Plasmonic and sensing; analytical devices (SERS)	[54]
Flower-like	200–300	Wet-chemical method	Analytical devices (SERS); catalysis	[54,65,66]

**Table 2:** Correlation between the coating nature and AgNPs stabilization mode.

Shape	Type of Coating	Size (nm)	AgNPs Stabilization	Ref.
Spherical	Citrate	14–20	Electrostatic	[105]
Triangular	Citrate	10–20	Electrostatic	[98]
Spherical	Plant root extract	30–55	Electrosteric	[109]
Spherical	Sodium dodecyl sulfate (SDS)	26	Electrosteric	[110]
Spherical	Tween 80	17–42	Steric	[110]
Nanocube	PVP	80	Steric	[111]
Nanobeam	PVP	17–70	Steric	[111]
Triangular	Chitosan	115–123	Steric	[91]
Spherical	PVA	8–46	Steric	[55]
Spherical	Silica	55–65	Electrostatic	[112]
Triangular	Silica	40–50	Electrostatic	[113]

**Table 3:** Summary of the main Ag-Au bimetallic NPs structures: synthesis techniques, size and shape.

Alloy/Core@shell	Shape	Synthesis Technique	Size (nm)	Ref.
AgAu alloy	Spherical	Chemical co-reduction of HAuCl_4_ and AgNO_3_ with sodium citrate	10–25 (0.27 < %Au < 1.00)	[144]
AgAu alloy	Spherical	Simultaneous laser ablation of Ag and Au colloids	5–50	[145]
AgAu alloy	Spherical	Metal evaporation on glass support and annealing (500 °C)	20–50	[146]
AgAu alloy	Spherical	UV laser radiation (193 nm) on silicate glass	5–40	[147]
AgAu alloy	Spherical core/alloy/shell	Sodium citrate reduction of Ag^+^ on AuNPs and hydrothermal treatment	16–26 (T = 120 °C) 16–23 (T = 160 °C)	[148]
AgAu alloy	Elliptical (quasi-spherical)	Metal evaporation on glass support and annealing (350 °C)	Vertical radius: 4–12 Horizontal radius: 6–15	[149]
AgAu alloy	Nanoprisms (NPrs)	Nanosphere lithography and film deposition by thermal evaporation on a glass	150 (length) 50 (height)	[150]
Ag@Au core@shell	Spherical	Laser ablation of Au in a suspension of Ag colloids	30 (Ag core) 0.5–4 (Au shell)	[53]
Ag@Au core@shell	Nanoplates (NPls)	Electrodeposition of Au shell on Ag nanoplates (AgNPls)	50 (Ag core) 0.5 (Au shell)	[151]
Ag@Au core@shell	Hemispherical NPls	Cycles of electrodeposition of Au shell on AgNPls supported on ITO glass	100 (Ag core: width) 40 (Ag core: height) 1 (Au shell–20 cycles)	[152]
Ag@Au core@shell	Triangular nanoprisms (TNPrs)	Chemical reduction of HAuCl_4_ by AA with PVP on silver TNPrs (AgTNPrs) by slow addition of HAuCl_4_ solution	60 (Ag core) 1 (Au shell)	[153]
Au@Ag core@shell	Spherical	Deposition of Ag (chemical reduction) on AuNPs	10–15 (Au core) 1–10 (Ag shell) 30 (Au core)1–9 (Ag shell)	[40]
Au@Ag core@shell	Nanorods (NRs)	Sodium citrate and AA reduction of AgNO_3_ on AuNRs	35 (Au core: length) 10 (Au core: width) 1–6 (Ag shell)	[154]
Au@Ag core@shell	NRs	Chemical reduction of AgNO_3_ with AA on seed-mediated grown in NaBH_4_ on AuNRs	60 (Au core: length) 30 (Au core: width) 1–3 (Ag shell)	[155]
Au@Ag core@shell	NRs	Chemical reduction of AgNO_3_ with AA on seed-mediated grown in NaBH_4_ on AuNRs	60 (Au core: length) 20 (Au core: width) 4 (Ag shell)	[156]
Au@Ag(@Au) core@shell	TNPrs	Sodium citrate and AA reduction of AgNO_3_ on seed-mediated grown AuNPs supported on an ITO glass (followed by electrodeposition of a thin Au layer)	Initial Au@Ag TNPrs 30 (height) Au shell very thin when it is present	[157]

## Abbreviations

AA	Ascorbic acid
Ag	Silver
AgNO_3_	Silver nitrate
AgNPs	Silver nanoparticles
Ag@AuNPs	Silver gold core@shell nanoparticles
AgAuNPs	Silver gold alloy nanoparticles
APTMS	3-Aminopropyltrimethoxysilane
Au	Gold
AuNPs	Gold nanoparticles
BSA	Bovine serum albumin
CTAB	Cetyltrimethylammonium bromide
Cu	Copper
FDA	Food and Drug Administration
HAuCl_4_	Tetrachloroauric (III) acid
ITO	Indium tin oxide
LOD	Limit of detection
LSPR	Localized surface plasmon resonance
MEF	Metal-enhanced fluorescence
MOF	Metal-organic framework
MPTES	(3-Mercaptopropyl) triethoxysilane
NaBH_4_	Sodium borohydride
NaHS	Sodium hydrosulfide
Na_2_S	Sodium disulfide
NC	Nanocages
NIR	Near-infrared
NOM	Natural organic matter
NPls	Nanoplates
NPrs	Nanoprisms
NPs	Nanoparticles
NRs	Nanorods
NS	Nanoshells
NSL	Nanosphere lithography
ORG	Oxidation reduction growth
PEG	Poly(ethylene) glycol
PVA	Poly(vinyl acetate)
PVP	Polyvinyl pyrrolidone
RI	Refractive index
RIS	Refractive index sensitivity
RIU	Refractive index unit
SA	Streptavidin
SDS	Sodium dodecyl sulfate
SEM	Scanning electron microscopy
SERS	Surface-enhanced Raman spectroscopy
SIF	Silver island film
SHE	Standard hydrogen electrode
SPP	Surface plasmon polariton
SPR	Surface plasmon resonance
TEM	Transmission electron microcopy
TEOS	Tetraethyl orthosilicate
TNPls	Triangular nanoplates
TNPrs	Triangular nanoprisms
TOAB	Tetraoctylammonium bromide
VdW	Van der Waals
